# The Brain-Heart Connection in Takotsubo Syndrome: A Neurobiological Perspective

**DOI:** 10.31083/RCM48476

**Published:** 2026-05-25

**Authors:** Li Qi, Wenjia Wang

**Affiliations:** ^1^Department of Neurology, The First Affiliated Hospital of Anhui Medical University, 230022 Hefei, Anhui, China; ^2^Department of Psychology and Sleep Medicine, The Second Affiliated Hospital of Anhui Medical University, 230601 Hefei, Anhui, China; ^3^Anhui Province Key Laboratory of Cognition and Neuropsychiatric Disorders, 230032 Hefei, Anhui, China; ^4^Collaborative Innovation Center of Neuropsychiatric Disorders and Mental Health, 230032 Hefei, Anhui, China; ^5^Department of Neurology, The Second Affiliated Hospital of Anhui Medical University, 230601 Hefei, Anhui, China

We reviewed Dr. Y-Hassan’s [1] recently published paper that elucidates coronary artery changes in Takotsubo syndrome (TTS), which showed interesting findings regarding the constriction of microcirculation due to the effect of mechanical forces [1]. It is worth mentioning that TTS, for the first time, was also discussed with the occurrence of a coronary artery–left ventricular micro-fistulae (CALVMF). Some have undergone temporary compression in the early stages, then return after recovery of heart function. The findings reveal an association between cardiac structural dynamics and coronary blood flow. However, the review focuses primarily on explaining these changes as potential results of mechanical compression. While acknowledging the crucial role of sympathetic overactivity in TTS pathogenesis, its specific scope does not extensively detail the neurobiological pathways. Therefore, we have taken this opportunity to provide a supplementary perspective based on neurobiology for further consideration regarding the origin of the ischemia that causes TTS.

Current research shows that the heart–brain axis is significant for TTS [2]. This aligns with the vascular findings of the Y-Hassan study on TTS [1]. The gray matter volumes decrease in distinct areas of the brain in those suffering from TTS, as reported in magnetic resonance imaging (MRI) studies [3, 4]: in the amygdala, hippocampus, and cingulate gyrus. These reductions occur across multiple structural MRIs. In addition, researchers have reported reduced functional connectivity among the limbic and autonomic regulatory systems—most notably, the decreased connection between the sympathetic and parasympathetic systems [5].

Neurochemical modulation facilitates coronary microvascular dysfunction and also represents an additional mechanism that amplifies the mechanical impairment described by Y-Hassan [1]. Classic data demonstrate marked increases in plasma catecholamines above baseline during TTS and which surpass those seen during myocardial infarction (MI) [6]. Supraphysiological catecholamines cause intracellular calcium overload, oxidative stress, and, ultimately, myocardial damage via a direct pro-inflammatory effect [7, 8]. *In vivo*, this may result in a switch of β_2_-adrenergic receptor signaling from the Gs to the Gi pathway, leading to negative inotropy and apical stunning [9]. At the same time, intense sympathetic stimulation releases a co-transmitter called neuropeptide Y, which stimulates Y1 receptors and produces considerable vasoconstriction via this pathway [10]. This “sympathetic storm” is considered to be an important component of the pathophysiology of TTS [11].

These recent findings point to coronary anomalies of TTS originating from aberrant central nervous system signaling, neurochemical vasoconstriction, and mechanical compression working in concert. Bridging our current understanding of the psychoneurobiology and mechanics of the disease state is needed for a full picture of this syndrome [12] and underscores the overlap with neurogenic stunned myocardium [13].
